# Experimental huts trial of the efficacy of pyrethroids/piperonyl butoxide (PBO) net treatments for controlling multi-resistant populations of *Anopheles funestus s.s.* in Kpomè, Southern Benin

**DOI:** 10.12688/wellcomeopenres.14589.1

**Published:** 2018-06-13

**Authors:** Romaric Akoton, Genevieve M. Tchigossou, Innocent Djègbè, Akadiri Yessoufou, Michael Seun Atoyebi, Eric Tossou, Francis Zeukeng, Pelagie Boko, Helen Irving, Razack Adéoti, Jacob Riveron, Charles S. Wondji, Kabirou Moutairou, Rousseau Djouaka

**Affiliations:** 1 University of Abomey, Calavi, Abomey-Calavi, 526, Benin; 2 AgroEcoHealth Platform, International Institute of Tropical Agriculture, Cotonou, 0932, Benin; 3 National University of Sciences, Technologies, Engineering and Mathematics of Abomey, Abomey, 123, Benin; 4 Cell Biology and Genetics Unit, Department of Zoology, University of Ibadan, Ibadan, Nigeria; 5 Faculty of Sciences, Department of Biochemistry, University of Yaounde I, Yaounde, 812, Cameroon; 6 National malaria and Neglected diseases control program, Ministry of Health, Cotonou, Benin; 7 Liverpool School of Tropical Medicine, Liverpool, L3 5QA , UK

**Keywords:** An. funestus s.s., An. coluzzii, Pyrethroids, PBO, LLINs, Multi-resistance controlling

## Abstract

**Background: **Insecticides resistance in *Anopheles* mosquitoes limits Long-Lasting Insecticidal Nets (LLIN) used for malaria control in Africa, especially Benin. This study aimed to evaluate the bio-efficacy of current LLINs in an area where *An. funestus* *s.l.* and *An. gambiae* have developed multi-resistance to insecticides, and to assess in experimental huts the performance of a mixed combination of pyrethroids and piperonyl butoxide (PBO) treated nets on these resistant mosquitoes.

**Methods: **The study was conducted at Kpomè, Southern Benin. The bio-efficacy of LLINs against *An. funestus and An. gambiae* was assessed using the World Health Organization (WHO) cone and tunnel tests. A released/recapture experiment following WHO procedures was conducted to compare the efficacy of conventional LLINs treated with pyrethroids only and LLINs with combinations of pyrethroids and PBO. Prior to huts trials, we confirmed the level of insecticide and PBO residues in tested nets using high performance liquid chromatography (HPLC).

**Results: **Conventional LLINs (Type 2 and Type 4) have the lowest effect against local multi-resistant *An. funestus s.s. and An. coluzzii *populations from Kpomè. Conversely, when LLINs containing mixtures of pyrethroids and PBO (Type 1 and Type 3) were introduced in trial huts, we recorded a greater effect against the two mosquito populations (P < 0.0001). Tunnel test with *An. funestus s.s. *revealed mortalities of over 80% with this new generation of LLINs (Type 1 and Type 3),while conventional LLINs produced 65.53 ± 8.33% mortalities for Type 2 and 71.25 ±7.92% mortalities for Type 4. Similarly, mortalities ranging from 77 to 87% were recorded with the local populations of *An. coluzzii*.

**Conclusion: **This study suggests the reduced efficacy of conventional LLINs (Pyrethroids alone) currently distributed in Benin communities where *Anopheles* populations have developed multi-insecticide resistance. The new generation nets (pyrethroids+PBO) proved to be more effective on multi-resistant populations of mosquitoes.

## Introduction

Malaria is responsible for about 438,000 deaths with an estimated 214 million disease cases annually ^1^. Malaria vector control tools have been encouraging worldwide, resulting in a decreased morbidity and mortality as of 2016 compared to the 2000 ^2^. Unfortunately, as this disease has reduced globally, it has been a different case in Africa, where malaria is still a serious challenge ^2^. Long lasting insecticide-treated nets (LLINs) are major components of malaria control tools, and they have helped to combat malaria disease when in good conditions and properly used ^2^. LLINs are effective, simple to use, easy to deliver to rural communities, and cost-effective when used in highly endemic malaria areas ^3^.

In Benin, malaria control is hugely dependent on LLINs and indoor residual spraying (IRS) ^4, 5^. In October 2014, there was a country-wide distribution campaign of mosquito nets to ensure universal coverage, with the free distribution of 6,077,272 LLINs to 2,199,522 households surveyed ^6^. After this exercise, LLINs utilization by children under five rose from 70% in 2012, to 73% in 2014 ^6^. However, the emergence and spread of resistant malaria vectors to insecticidal components used for treating these nets have threatened the earlier progress made with this malaria vector control tool ^7– 9^. Resistance to insecticides ofone of the main malaria vectors, *An. funestus* against control tools has since become a serious challenge facing the quest for malaria elimination in Africa. Reported cases of resistance are available in countries such as Cameroon ^10^, Uganda ^11^, Mozambique ^12^, Malawi ^13, 14^, Ghana ^15^, Nigeria ^16^ and Benin ^17, 18^. There are also multiple mechanisms that are driving observed resistance in this mosquito population, although over-expression of detoxification genes remains the main driving force of insecticide resistance in this *Anopheles* species ^15, 19^. Another observation is that resistance mechanisms are known to differ from one mosquito population to another suggesting the contribution of geographical differences in resistance profiling ^15, 20^. This revelation is of serious concern because it is becoming a significant threat to existing malaria control tools. Recently, a study by Agossa *et al*. ^9^ in the northern part of Benin, showed that the efficacy of existing malaria vector control tools treated with pyrethroid have decreased in wild *An. gambiae s.l*. populations. Considering the fact that *An. funestus* and *An. gambiae* have developed resistance to almost all classes of insecticides across Benin ^17, 21, 22^, it might follow a similar trend as the above study. Indeed, there is a serious quest for alternative insecticides since pyrethroids are becoming less effective with recorded reports of resistance in malaria vectors ^23^. Pyrethroids are very safe, acceptable and suitable for LLINs, but degrade very fast, especially when exposed to sunlight, which can be avoided if nets are well preserved ^24^. A different insecticide resistance management approach combining a chemical synergist, piperonyl butoxide (PBO), with pyrethroids on net fibres could be a promising way to fight insecticide resistance. PBO, a synergist capable of inhibiting the action of oxidase enzymes, has potential to combat the growing problem of oxidase based pyrethroid resistance in mosquito vectors species. Two types of long lasting nets treated with permethrin+PBO and deltamethrin+ PBO are the new generation of LLINs for improved resistance management ^25, 26^. These new generation nets have shown their efficacy on some resistant populations of *Anopheles* in experimental hut trials ^25, 27^. In hut trials, the new generation of LLINs increases mortality and inhibits blood feeding against pyrethroid-resistant *An. gambiae* in some Africa regions ^23, 25, 26, 28^. In Nigeria, the efficacy of LLINs treated with deltamethrin +PBO was highly effective on resistant *An. gambiae* compared with standard treated nets with no PBO ^29^. Also, in Southern Africa (Mozambique), this combination proved to be more effective against resistant *An. funestus* and *An. gambiae* ^12, 20^.

Due to the widespread of insecticide resistance in most populations of *An. funestus* from South to the North of Benin ^17, 18, 21^, it is important to assess the efficacy of currently used LLINs (conventional LLINs) and also conduct in experimental huts a comparative assessment of the performance of the new generation of treated LLINs (Pyrethroids+PBO) against conventional LLINs currently used by communities in areas of Benin where the main malaria vectors *An. gambiae* and *An. funestus* have developed multiple resistance to insecticides.

## Methods

### Study area

The assessment was conducted in the rural locality of Kpomè (6° 23¢ N, 2° 13¢E) located in South Benin, approximately 81 km from Cotonou. The study area has a sub-tropical climate, receives 1,100 mm of mean rainfall annually and has a mean monthly temperature between 27 and 31°C. The rainfall pattern in this area is similar to other southern localities of Benin, with two rainy seasons and two dry seasons. The constant presence of water bodies in this locality favors the development of *An. funestus* and other mosquito species ^18^. Previous studies carried out in Kpomè showed that *An. funestus s.s.* is mainly predominant during the dry season and transitional periods, and exhibited high resistance to permethrin and deltamethrin with mortalities rates ( World Health Organization (WHO) susceptibility tests) of 13% and 46.5% respectively ^17^. P450s are the main family of detoxification enzymes involved in observed pyrethroid resistance in the *An. funestus* population in this locality ^17^. *An. gambiae* populations from this same locality have also developed multi-resistance to several insecticides families ^30^. This set of available environmental and entomological data has prompted building of seven experimental huts at Kpomè for trials to identify best LLINs types for improved control of insecticide resistant populations of malaria vectors.

### Collection of mosquitoes for planed experiments

Early morning collections of blood-fed, semi-gravid and fully gravid females of resistant *An. funestus* resting inside houses were collected using electric aspirators between 06h00 and 10h00 in June 2017 (Consent from head of household was obtained prior to collection). These mosquitoes were identified morphologically using Gillies and De Meillon ^31^ and Gillies and Coetzee ^32^ key as *An. funestus* were kept in small cups and immediately transported to the laboratory (Relative humidity of 70–80% and a temperature of 25–30 °C) until fully gravid (for blood-fed and semi-gravid females). Eggs were obtained from F _0_generation (Collected females from the field) using the forced egg laying method ^33^ and were allowed to hatch to obtain F _1_ generation to be used for different experiments.

Still in the same locality, *An. gambiae* breeding sites were surveyed and their larvae collected using dipping methods ^34^ and reared in the insectary to adult stage for different experiments.

### Species characterization for *An. funestus* and *An. gambiae*

A subset (100 *An. funestus* and 100 *An. gambiae*) of mosquitoes to be used for various assays were subjected to molecular speciation prior to assays. DNA of *An. funestus s.l.* used for forced-laying was extracted using the Livak method ^35^. Species-specific targets were amplified for DNA using method described by Koekemoer *et al.* ^36^, before distinct separations on a 1.5% agarose gel electrophoresis. For *An. gambiae,* we used also the protocol of Livak ^35^, for DNA extraction followed by an amplification based on the protocol described by Fanello *et al*. ^37^ then after, amplified products were migrated on agarose gel for describing banding patterns.

### Insecticide susceptibility pattern of mosquito populations used in this study

Prior to assays, a subset of *An. funestus* and *An. gambiae* were subjected to insecticide susceptibility tests to confirm resistance levels of collected *Anopheles* species from Kpomè. Similarly, *An. gambiae Kisumu* were also tested to confirm their susceptibility. Unfed F1 *Anopheles* female mosquitoes (2–5 days old) were therefore tested with 2 insecticides: 0.75% permethrin (type I pyrethroid) and 0.05% deltamethrin (type II pyrethroid) by using WHO susceptibility tests ^38^. Approximately 100 mosquitoes (4 replicates of 25 mosquitoes) were used per test. The knockdown rate of mosquito exposed to the insecticides was recorded each 5 min, during 1 h exposure-period. A 10 % of sugar solution was made available to survivors. This test was made under observation at 25°C and 80% relative humidity laboratory condition. Mortality was recorded 24h after exposure to each insecticide. According to WHO criteria ^38^, vectors were considered as being susceptible to a given insecticide if mortality rate was ≥ 98 %, resistant if mortality was <90 % or possibly resistant if mortality was between 90 and 98 %.

### Piperonyl butoxide (PBO) synergist tests

According to the level of observed resistance against permethrin and deltamethrin, and because of pyrethroids resistant, *An. funestus* population has been shown to express P450s genes more than in previous studies ^19, 39, 40^, 2–5 days old F1 mosquitoes were pre-exposed to 4% PBO paper for 1 h and immediately exposed to 0.75% permethrin and 0.05% deltamethrin for 1 h. Two controls were used during this experiment. The first control was the mosquitoes exposed to untreated papers without PBO, and the second comprised of mosquitoes exposed to paper treated with PBO only. Mortalities were recorded 24h post exposure and were later compared to the un-synergized group in order to evaluate the potential role of cytochromes P450 genes in the observed resistance.

### Characteristics of long-lasting insecticidal nets used during the various assessments

Five types of LLINs were used for the phase I (Cone and tunnel tests) and phase II (experimental hut) evaluations.

The Type 1 LLINs made of monofilament polyethylene (100 mesh size) fabric treated with deltamethrin at 4 g/kg±25% and piperonyl butoxide (PBO) at 25g/kg±25%, side panels made of multifilament polyester fabric with a strengthened border treated with deltamethrin at 2.1 g/kg±25%.

The Type 2 LLINs was made of multifilament polyester fabric (100 mesh size), treated with a deltamethrin only (no PBO added) at 1.4 g/kg±25%.

The Type 3 LLINs was treated with 20 g/kg of permethrin and 10 g/kg of PBO in the whole polyethylene net fibres (150 mesh size).

The Type 4 LLINs made of polyethylene fibers treated with permethrin only (no PBO) at 20 g/kg, incorporated during fibers extrusion (150 mesh size).

The Type 5 was an untreated net, a multifilament polyester (100 mesh size) fabric with neither insecticide nor PBO treatments.

All nets used had sizes of 160 cm wide, 180 cm long and 150 cm high. All types of nets treated and control nets were procured by the Liverpool School of Tropical Medicine (LSTM), UK, properly wrapped and shipped to us at IITA for our various trials.

### WHO cone tests with of 5 types of nets under tests

Four cones were fixed in contact to 25 × 25 cm pieces of nets taken from the sides and top panels of LLINs (Methods in *Anopheles* Research, 2010). 2 to 5 days old individuals from the 3 colonies of *Anopheles* mosquitoes (resistant- *An. funestus* Kpomè, Resistant- *An. gambiae* Kpomè and Susceptible- *Anopheles Kisumu*) were exposed to nets for 3 min, after which they were transferred into recovery paper cups and provided with cotton wool soaked in a 10 % honey solution. A minimum of 50mosquitoes was tested for each net. At least three pieces per net was used for this test. Mosquito knock-down rate (kd) was recorded at 1h post-exposure period and the mortality rate was determined 24 h post-exposure. The mortality rate was corrected using Abbott’s formula if needed. These tests were conducted at a room temperature and relative humidity of 25–30°C and 70–80% respectively.

### WHO tunnel tests with fragments of the 5 types of nets under tests

Tunnel test was carried out with the same samples of LLINs used for cone test. Adults *An. funestus, An. gambiae* and *Kisumu* strain were also used for this test ^41^. Adult mosquitoes aged between 5 to 8 days were released at 6.00 PM in the first compartment C1 of a 60-cm long tunnel made of glass divided by a transverse netting (25cm x 25 cm) insert, fitted onto a frame that slots across the tunnel. The LLINs fragments used had been pierced (1-cm diameter holes) to allow mosquitoes to pass through it into the tunnel to the compartment C2 where a guinea pig was placed for mosquitoes feeding. Each guinea pig was used only once for this study. Guinea pigs were sourced from local markets where they are sold for food consumption. At 8.00 AM of the following morning, mosquitoes were collected from both compartments and transferred into plastic cups. The mortality and feeding status (blood-fed or unfed) of each mosquitoes collected from the tunnel were recorded. Blood-feeding rate and penetration rate across the tunnel were also assessed.

### Experimental hut trials of the efficacy of the 5 Net Types on insecticide resistant mosquitoes

Experimental huts newly built in Kpomè are specially designed to test the efficacy of different vector control products against freely entering mosquitoes under natural but controlled conditions. This facility was used for our release and recaptures tests. Huts were typical of the West African model as recommended by WHO ^41^. The 3.5 × 2 × 2 m huts were made from concrete bricks, with a corrugated iron top and a ceiling of thick polyethylene sheeting lined, and each was built on a concrete base surrounded by a water-filled moat to exclude ants. Mosquito access was through 4 window slits, constructed from pieces of iron fixed at an angle to create a funnel with a 1-cm gap, present on 3 sides of the huts. Mosquitoes had to fly upward to enter through the gaps and downwards to exit; this precluded or limited exodus through the aperture and enabled us to account for most entering mosquitoes. A veranda trap made of concrete bricks and mesh screening (2 m long × 1.5 m wide × 1.5 m high) projected from the back wall of each hut. Movement of mosquitoes between a room and the veranda was unimpeded.

### Study design

The described 5 types of mosquito nets were assessed against pyrethroid resistant *An. funestus and An. gambiae.* The control mosquito population used was only *An. gambiae Kisumu* as we had neither laboratory/field susceptible *An. funestus*, nor field susceptible *An. gambiae*.

The following five comparison arms were tested in separate huts:

1- LLINs Type 1 (deltamethrin + PBO)

2-LLINs Type 2 (deltamethrin only)

3-LLINs Type 3 (permethrin + PBO)

4-LLNIs Type 4 (permethrin only)

5-Type 5 Untreated Polyester net (control)

### Blank assessment of hut attractiveness

Prior to introducing nets into huts, we conducted preliminary experiments which showed the huts to be evenly attractive to mosquitoes. Briefly, assessment for freely-entered mosquitoes in the hut was conducted during 2 weeks and the attractiveness effects of each hut were evaluated. Adult male volunteers slept under the untreated net in the huts from 20:00 hours to 05:00 hours each night after cleaning the hut to remove any spiders and ants. To minimize biases in individual attractiveness, sleepers were rotated between huts on successive nights throughout the 2 weeks.

### Blank assessment of huts lethality

Still prior to assessments, an initial series of bioassays was conducted to determine the mortality of susceptible mosquitoes exposed to various surfaces in the huts to know the lethal effect of the huts. Bioassays were performed with WHO cones tests attached to the surfaces with masking tape. In each hut, surfaces tested included doors, walls, screening-mesh of veranda, ceiling and floor. Ten females of *An. gambiae Kisumu* strain of 2 to 5 days old were put into each cone for 30 min. After this exposure time, they were removed from the cone and put into plastic cups covered with untreated mosquito net and given access to 10% honey solution and mortalities recorded after 24h.

### Release and recapture experiment

A release/recapture experiment was conducted in experimental huts with resistant populations of *An. funestus and An. gambiae* both from Kpomè and a susceptible *An. gambiae Kisumu*. These 3 populations of mosquitoes were released different days into huts where the 5 described types of nets were erected. The experiment was conducted as described by WHO protocols ^41^. The main trial was conducted in August 2017. The treatments were allocated randomly to five experimental huts in study site. Each net was deliberately holed with six 4cm×4cm holes to simulate a worn net. Before experimental hut evaluation, adult volunteers had been recruited among the inhabitants of the villages where experimental huts were implemented and informed consent to participate in the study was given beforehand and, chemoprophylaxis was provided during the trial. Female mosquitoes aged 5 days were released in each hut at 20:00 h in the night and monitored till morning. Early in the morning, released mosquitoes were recaptured from the hut, veranda and inside the nets and were scored as dead or alive and as fed or unfed. Live mosquitoes were kept in small cups containing sugar solution for 24 hours to assess the delayed mortality. Entomological effects of treatments were compared in-between nets and with the untreated net (control Net Type 5). Target entomological parameters monitored included: induced exiting, blood-feeding inhibition and mortality.

(i) insecticide-induced exiting, i.e. the proportion of mosquitoes found in hut verandahs relative to control huts; (ii) blood-feeding inhibition, i.e. the proportional reduction in blood-feeding relative to untreated nets; and (iii) mortality, the proportion of mosquitoes killed (immediate plus delayed).

### Chemical analysis of net used in the experimental hut trial

Prior to the trial, chemical analysis were conducted on pieces of nets (pieces from holes made on nets) from the five Net Types erected in each hut. This experiment was to confirming the presence, or absence, and the concentration of pyrethroids and PBO in each net to be used in this trial. For the LLINs Type 1, the side panels and top panel were tested separately. Chemical analysis was conducted using high performance liquid chromatography (HPLC) machine (Agilent technology1260 infinity, Germany). Deltamethrin, permethrin and PBO was extracted using acetonitrile as solvent and the mixture was sonicated for 15min. Afterwards, the solution without the net was transferred into a new flask and filtered through a 0.45µm PTFE syringe filter into an HPLC vial for analysis. For HPLC analysis, standard solutions of each insecticide (Permethrin cat no. 45614, Deltamethrin cat no.45423 and PBO cat no.45626) purchased from Sigma Aldrich were prepared from stock solution in acetonitrile. Standard curve of each insecticide were drawn. The HPLC system condition was as follow: mobile phase: Acetonitrile /H2O (90:10), C18 Column, flow rate: 1ml/min, injection volume: 50µl and UV detector wavelength: 226nm.The quantities of insecticides were calculated based on the peak area and expressed in g/kg of net.

### Data analysis

Data from bioassays were compared between each net using MedCalc easy-to-use online statistical software, version 18.2.1 ^42^, while the Fisher’s exact test was used to test for significant difference of mortality rates. Significance between treatments was set at 5% level. The proportion of mosquitoes that exited early (induced exophily), the proportion that was killed within the hut (mortality) and the proportion that successfully blood-fed (blood feeding rate) were compared and analyzed using the logistic regression with treatments as fixed effects and huts, sleepers as random effects ( STATA 9 Software).

## Results

### Molecular speciation of *An. funestus* and *An. gambiae*

All 100 samples of *An. funestus* and 100 samples of *An. gambiae* from Kpomè analyzed for molecular speciation were *An. funestus s.s.* and *An. coluzzii* respectively ( Table 1).

**Table 1.** Distribution of members of *Anopheles* groups collected in June 2017 in Kpomè, Southern Benin.

Mosquito species	*Anopheles* subjected to molecular speciation	*An. funestus s.s.*	*An. coluzzii*
*An. funestus s.l.*	100	100	/
*An. gambiae s.l.*	100	/	100

### Insecticide susceptibility profiles of *Anopheles* populations used in this study

**Figure 1.**
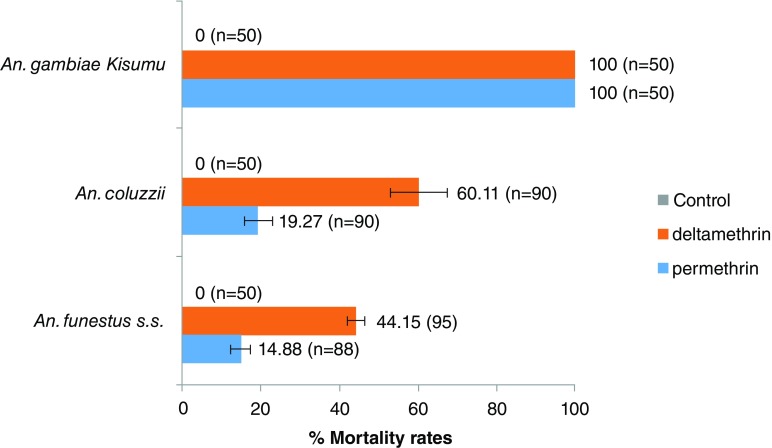
Insecticide susceptibility profiles of *Anopheles* to permethrin and deltamethrin. Error bars represent standard error of the mean.

*An. gambiae Kisumu* was used in this test as a control for checking the quality of impregnated papers. A mortality rate of 100% was recorded with permethrin and deltamethrin treated papers. With local populations of *An. funestus s.s.* we recorded 24 h post-exposure, mortalities of 14.84±2.32% and 44.15±2.23% for permethrin and deltamethrin respectively ( Figure 1). Low mortality rates were also recorded for *An. coluzzii* population from Kpomè against permethrin (19.27±3.52%) and deltamethrin (60.11±7.19%). No mortality was recorded when subsets of these mosquitoes species were exposed to papers with no insecticides (Control).

### Synergist assay with PBO

**Figure 2.**
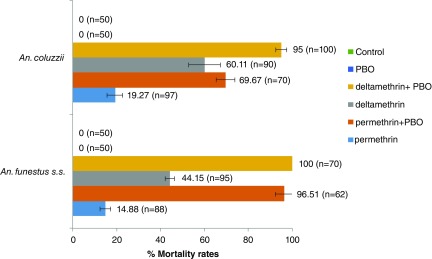
Insecticide susceptibility profiles of *Anopheles* to pyrethroids when combined with piperonyl butoxide (PBO). Error bars represent standard error of the mean.

When permethrin and deltamethrin were combined with PBO ( Figure 2), mortalities in *An. funestus s.s.* rose from 14.84% to 96.51% (permethrin) and from 44.15% to 100% (deltamethrin). However with *An. coluzzii* population, mortalities reached 95% with deltamethrin + PBO whereas, the combination permethrin + PBO lifted up the mortality from 19.27% to 69.67%.

### Chemical analysis of insecticide contains of nets used in the experimental hut trial

HPLC analysis conducted on net fiber showed that deltamethrin concentration in the side of the Net Type 1(1.570±0.024 g/kg) and in the roof (3.762±0.019g/kg) were all within the standard dose (2.1g/kg±25% on sides and 4g/kg ±25% in the roof) ( Table 2).A mean dose of insecticide from the five sides of treated Net Type 2(0.994±0.013g/kg) and treated net Type 3 (16.065±0.244 g/kg) analyzed using HPLC were closer to the standard doses (1.4g/kg ±25% and 20g/kg ±25% respectively) as recommended by manufacturers. Similarly for Type 4, the permethrin concentration for sides/top panels (23.702±0.003 g/kg) was within the standard dose. No presence of insecticides traces on the untreated net (Type 5) was revealed by this HPLC analysis.

**Table 2.** Concentrations of insecticides and synergist in the net fragments when analyzed by HPLC techniques.

Net Types	Net sections	Chemicals	Units	Standard concentrations	Recorded concentrations
**1**	Sides	deltamethrin	g/Kg	2.1 ± 25%	1.570 ± 0.024
	Roof	deltamethrin	g/Kg	4 ± 25%	3.762 ± 0.019
		PBO	g/Kg	25 ± 25%	26.210 ± 0.057
**2**	Sides/Roof	deltamethrin	g/Kg	1.4 ± 25%	0.994 ± 0.013
**3**	Sides/Roof	permethrin	g/Kg	20 ± 25%	16.065 ± 0.244
	Sides/Roof	PBO	g/Kg	10 ± 25%	11.016 ± 0.003
**4**	Sides/Roof	permethrin	g/Kg	20 ± 25%	23.702 ± 0.003
**5**	Sides/Roof	deltamethrin	g/Kg	0	0
	Sides/Roof	permethrin	g/Kg	0	0
	Sides/Roof	PBO	g/Kg	0	0

PBO - piperonyl butoxide, HPLC - high performance liquid chromatography

### WHO cone tests with the 5 types of nets

**Figure 3.**
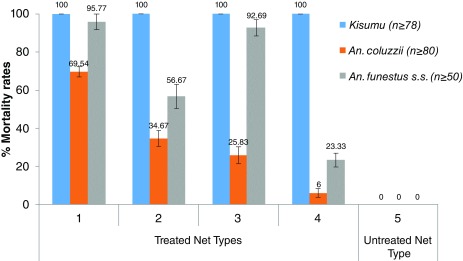
Cone tests performed on different net Types with using pyrethroids resistant *Anopheles funestuss.s. a*nd *An. coluzzii*. Error bars represent standard error of the mean.

A total of 275, 461 and 462 females of *An. funestus s.s*., *An. coluzzii* and *Kisumu* strain respectively aged 2-5 days were exposed to the five sides of nets following WHO standard cones protocol. No mortality was recorded with mosquitoes exposed to untreated Net Type 5 after 24 hrs. *An. gambiae Kisumu* showed full susceptibility to all treated net. The Net Type 2 had the lowest lethal effect on both resistant *An. funestus s.s.* (56.67±6.38%) and *An. coluzzii* (34.67±4.11%) from Kpomè ( Figure 3). However, when they were exposed to Net Type 1 containing PBO + deltamethrin, the mortality rate rose from 56.67 % to 95.77% for *An. funestus s.s* and a mean mortality of 69.54 ± 2.66% was recorded for *An. coluzzii* population showing that Net Type 1 is more effective against these *Anopheles* species compared to Net Type 2 (P < 0.0001).

A similar trend was observed when *An. funestus s.s.* were exposed to Net Type 4. Recorded mortality rate with Net Type 4 was 23.3 ± 3.59 %. When PBO was added into the net fibers for Net Type 3 (PBO + permethrin), a significant increase of the mortality rate was recorded (mortality rate with Net Type 3 = 92.69 ± 3.59%; *χ2* = 52.352, P < 0.0001).In contrast, when *An. coluzzii* mosquitoes from Kpomè were introduced into the various cones, mortalities slightly rose from 6% to 25.83% with Net Type 3.

### WHO tunnel tests with fragments of the 5 types of nets

When the three mosquito populations ( *An. gambiae Kisumu*, *An. funestus s.s. and An. coluzzii)* were separately released in tunnels with the untreated Net Type 5, we globally recorded over 45% penetration rates for all 3 mosquito species. When the untreated net was replaced by Net Type 2 and Net Type 1, the penetration rate decreased respectively from 85.66% to 3.4% and 0% for *An. gambiae Kisumu* ( Table 3). For the resistant *An. funestus s.s.*, we recorded an inhibition of the penetration in Net Type 2 (9.32%) and with side sections of Net Type 1 (1.79%) when compare to the untreated Net Type 5 (66.85%). No *An. funestus s.s.* was able to pass through the top section of the Net Type 1. When the pyrethroid resistant *An. coluzzii* population was released in tunnels containing fragments of treated Net Type 2 (deltamethrin only), side and top sections of Net Type 1, we recorded respectively penetration rates of 9.66±2.84%, 7.84±0.86% and 3.66 ±1.22% in the second compartment (C2) of tunnels. With fragments of Net Type 4 (bigger mesh size and treated with permethrin only), we recorded a similar penetration rate as with the control untreated Net Type 5 in resistant population of *An. funestus s.s.* However remarkable decreased penetration of this mosquito species through the Net Type 3 (PBO+permethrin) was observed. With Net Type 4 containing only permethrin and Net Type 3 containing permethrin+PBO, we recorded limited entry of *An. coluzzii* into compartment C2; 16.07±4.44% and 55.52±1.24% penetration rates respectively.

**Table 3.** Bio -efficacy of Long-Lasting Insecticidal Nets (LLIN) against resistant *Anopheles funestus s.s. and An. coluzzii* and laboratory susceptible ‘ *Kisumu*’ strain in tunnel test Standard errors are 95% confidence interval.

Species	Mosquito net Types	Released number	% Penetrating	% Blood feeding	% Blood feeding inhibition	% Overall mortality
***Susceptible*** ***An. gambiae*** ***(Kisumu)***	1 (Top panel)	92	0	0	100	100
	1 (Side panel)	97	0	0	100	100
	2	99	3.40 (±1.73)	0	100	99.17 (±0.83)
	3	93	1.22 (±1.22)	0	100	100
	4	100	11.10 (±5.22)	1.02 (±1.02)	98.9	100
	5	100	85.66 (±3.43)	91.92 (±0.81)	/	0
***Resistant*** ***An. coluzzii***	1 (Top panel)	82	3.66 (±1.22)	0	100	87.80 (±2.44)
	1 (Side panel)	89	7.84 (±0.86)	6.67 (±2.02)	80.30	63 (±2.12)
	2	92	9.66 (±2.84)	9.66 (±2.84)	71.48	56.82 (±6.82)
	3	82	16.07 (±4.44)	12.22 (±0.60)	63.92	77.88 (±3.52)
	4	72	55.52 (±1.24)	30.58 (±0.85)	9.71	45.91 (±2.66)
	5	77	45.65 (±6.07)	33.87 (±10.96)	/	0
***Resistant*** ***An. funestus s.s.***	1 (Top panel)	50	0	0	100	98 (±2)
	1 (Side panel)	51	1.79 (±1.79)	1.79 (±1.79)	97.66	92.47 (±3.18)
	2	51	9.32 (±4.97)	6.13 (±2.56)	91.98	65.53 (±8.33)
	3	53	5.57 (±1.57)	0	100	96.21 (±0.21)
	4	54	66.67	44.58 (±1.25)	41.75	71.25 (±7.92)
	5	51	66.85 (±9.15)	76.54 (±3.46)	/	0

A high blood feeding rate was observed in the insecticide resistant *An. funestus s.s.* population (76.54±3.46%), the resistant *An. coluzzii* (33.87±10.96%) and *Kisumu* strains (91.92±0.81%) released in tunnel containing untreated Net Type 5 ( Table 3). However, no *Kisumu* was able to blood feed in the presence of Net Type 1, Net Type 2 and Net Type 3. Only a single mosquito ( *Kisumu*) was able to blood feed in the presence of Net Type 4 containing permethrin and having large mesh sizes. Generally, all treated nets provided more blood feeding inhibition with *An. funestus s.s.* compare to *An. coluzzii*. Indeed, blood feeding was inhibited at respectively 91.98%, 97.66% and 100% with Net Type 2, Net Type 1(Side) and Net Type 1 (Top) in *An. funestus s.s.* In *An. coluzzii* population, Net Type 2, Net Type 1 (Side) and Net Type 1 (Top) provided respectively 71.48%, 80.30% and 100% blood feeding inhibition rates. Against resistant *An. funestus s.s.*, blood feeding inhibition rates with Net Type 4 (41.75%) was significantly lower than Net Type 4 (100%). Respectively 9.71% and 63.92% blood feeding inhibition rates were obtained with Net Type 4 and Type 3 in *An. coluzzii*.

Furthermore, lethal effect of all LLINs ranged from 99% to 100% against susceptible *An. gambiae Kisumu* strain. However, the mortality rate recorded in resistant *An. coluzzii* population with Net Type 2 increased when we exposed this mosquito species to Net Type 1. The same observation was noted with Net Type 2 and Type 1 (side and top sections) against resistant *An. funestus s.s.* where mortality rate rose from 65.53% to 92.47% and to 98% respectively. Less than 80% mortality was recorded in presence of Net Type 4 against *An. funestus s.s*., while 96% of mortality rate with Net Type 3. High lethal action was provided by Net Type 3 (77.88%) against *An. coluzzii* ( Table 3) compared to the Net Type 4(45.91%). Zero mortality was recorded with untreated Net Type 5 against the three *Anopheles* mosquito populations.

### Blank assessment of experimental huts attractiveness

A total of 603 mosquitoes were allowed to freely enter the seven experimental huts during the 12 trial nights. The mean number of mosquitoes collected in huts was high in hut N°7 (18.41) followed by hut N°2 (8.41). The mean number of mosquito per night was almost similar in huts N° 6, 5, 4 and 3. The hut N°1 showed a relatively low attractiveness ( Table 4). However, similar attractiveness in terms of *Anopheles* mosquitoes was observed between the hut N° 1, 2 and 5. The recorded mean numbers of *Anopheles* mosquito in hut N° 3, 4, 6 and 7, which are similar, were higher than the others.

**Table 4.** Mean number of mosquitoes collected per night in the seven experimental huts over 12 nights prior to phase II evaluation. Standard errors are 95% confidence interval.

Hut number	Overall mosquitoes	*Anopheles*
**1**	1.91(1.5 – 2,32)	0.33 (0.19 – 0.47)
**2**	8.41 (6.93 – 9.89)	0.5 (0.27 – 0.73)
**3**	4.16 (3.54 – 4.78)	0.83 (0.46 – 1.2)
**4**	6.83 (6.05 – 7.61)	1.25 (0.82 – 1.68)
**5**	4.83 (4.41 – 5.25)	0.67 (0.25 – 1.09)
**6**	5.67 (4.46 – 6.88)	1.17 (0.75 – 1.59)
**7**	18.41 (15.66 – 21.16)	1.25 (0.8 – 1.7)

### Blank assessment of experimental huts lethality

The cones bioassay conducted on various surfaces, such as doors, walls, screening-mesh of veranda, ceiling and floor, of each hut revealed that all the huts built in Kpomè locality had no lethal effect on susceptible *Anopheles gambiae Kisumu* strain. The mortality rate for all exposed mosquitoes was very low as only one mosquito died out of the total of 73 exposed in hut N°4 and N°7 ( Table 5).

**Table 5.** Lethal effect of experimental huts built at Kpomè, Southern Benin.

N° of experimental huts	Tested *An.* *gambiae Kisumu*	Dead mosquitoes	Mortality rates (%)
**Hut 1**	**73**	0	0
**Hut 2**	**76**	0	0
**Hut 3**	**79**	0	0
**Hut 4**	**73**	1	1.37
**Hut 5**	**73**	0	0
**Hut 6**	**74**	0	0
**Hut 7**	**73**	1	1.37

### Release and recapture experiments

**Figure 4.**
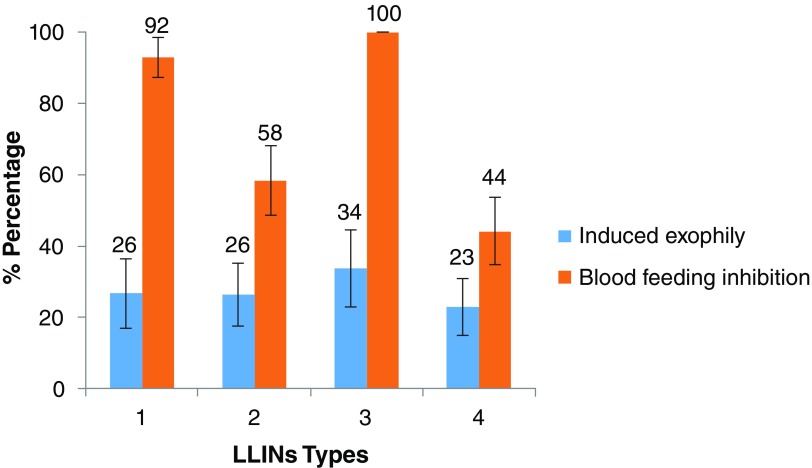
Induced exophily and blood feeding inhibition rates of resistant population of *Anopheles funestus s.s.* in experimental huts with selected treated nets. Error bars represent 95 % confidence interval. LLIN - Long-Lasting Insecticidal Nets.

**Figure 5.**
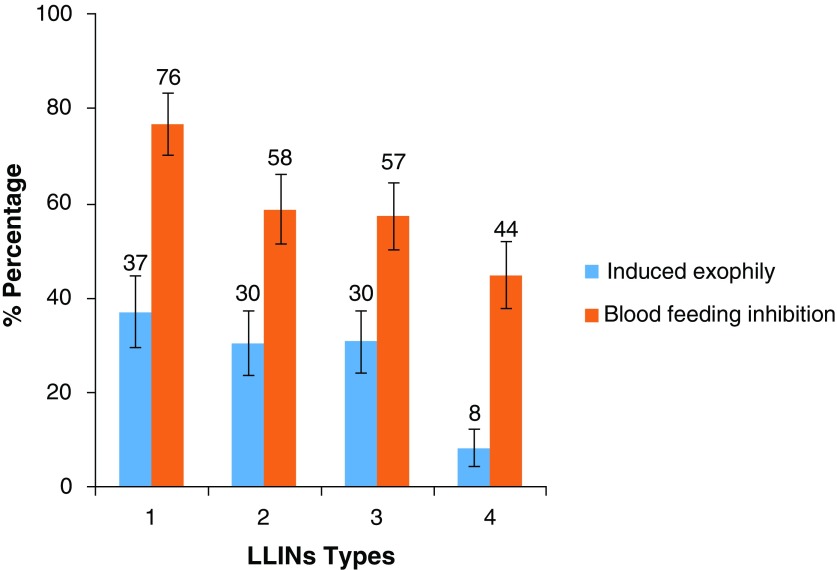
Induced exophily and blood feeding inhibition rates of resistant population of *Anopheles coluzzii* in experimental huts with selected treated Nets. Error bars represent 95 % confidence interval. LLIN - Long-Lasting Insecticidal Nets.

***Induced exophily***. When *Kisumu* strain was released in rooms containing treated nets, we recorded a significant movement of mosquitoes from the room to the veranda; all treated nets induced significant exophily rates ranging from 50 to 73% compared to the untreated net, where the observed exophily was 30% (P< 0.0009). A similar trend was observed with the pyrethroids resistant *An. funestus s.s.* population from Kpomè with exophily rates ranging from 30 to 40% with treated nets compare to 9.46% with untreated Net Type 5 ( Table 6). The induced exophily rates recorded with *An. funestus s.s.* was not significant in between huts containing treated nets (induced exophily rate ranging from 23 to 34%) ( Figure 4). As for the pyrethroids resistant *An. coluzzii*, all treated nets induced exophily rates ranging from 8% to 37% ( Figure 5).

**Table 6.** Summary of release-recapture experiment with susceptible strain of *Anopheles gambiae* (Kisumu) and resistant *An. funestus* s.s. and *An. coluzzii in* experimental huts.

Mosquito nets types	Total recaptured	% Exophily	% Blood feeding	% Personal protection	% Overall Killing effect
***Anopheles gambiae Kisumu***					
1	138	73.91 (66.59 - 81.24	0	100	97.08
2	139	69.78 (62.15 - 77.42)	0	100	97.08
3	135	51.11 (42.68 - 59.54)	0	100	94.89
4	138	50 (41.66 - 58.34)	0	100	95.62
5	142	30.28 (22.72 - 37.84)	71.83 (64.43 - 79.23)	/	/
***Resistant Anopheles coluzzii***					
1	160	40 (32.41 - 47.59	10 (5.35 - 14.65)	80.24	73.65
2	175	33.71 (26.71 - 40.72)	17.71 (12.06 - 23.37)	61.72	28.49
3	191	34.03 (27.31 - 40.75)	18.32 (12.84 - 23.81)	56.79	72.58
4	190	12.63 (7.91 - 17.36)	23.68 (17.64 - 29.73)	44.44	10.75
5	189	4.76 (1.73 - 7.80)	42.86 (35.80 - 49.91)	/	/
***Resistant Anopheles funestus s.s.***					
1	80	33.75 (23.99 - 44.11)	3.75 (-0.41 - 7.91)	92.30	100
2	96	33.33 (23.90 - 42.76)	21.88 (13.61 - 30.14)	46.15	77.02
3	72	40.28 (28.95 - 51.61)	0	100	87.83
4	109	30.28 (21.65 - 38.90)	29.36 (20.81 - 37.91)	17.94	48.64
5	74	9.46 (2.79 - 16.13)	52.70 (41.33 - 64.08)	/	/

***Blood feeding***. No *Anopheles Kisumu* bite was recorded when volunteer sleepers spent nights under all treated nets (100% blood feeding inhibition). In contrary, when these nets were replaced with untreated nets (Net Type 5), 71% biting rates were recorded with *Anopheles Kisumu*, 52% with pyrethroids resistant *An. funestus s.s.* and 42% with resistant *An. coluzzii*.

More specifically there was blood-feeding inhibition with *An. funestus s.s.* populations in presence of Net Type 4 (44% blood feeding inhibition) and Net Type 2 (58% blood feeding inhibition) compared to untreated nets. Generally, higher blood-feeding inhibition rates were provided by Net Type 1 containing deltamethrin + PBO (92% blood feeding inhibition) than Type 2 containing deltamethrin only (P<0.0001). Similarly, blood feeding inhibition rates in huts with Net Type 3 containing permethrin + PBO (100% blood feeding inhibition) was higher than those with Net Type 4 containing permethrin alone ( Figure 4) ( Table 6). As for the pyrethroid resistant *An. coluzzii*, blood feeding was inhibited more with Net Type 1 which contains deltamethrin + PBO (76% blood feeding inhibition) than Type 2 which contains deltamethrin only (58% blood feeding inhibition). Respectively, 44% and 57% blood feeding inhibition rates were recorded in huts with Net Type 4 and Net Type 3 ( Figure 5).

**Figure 6.**
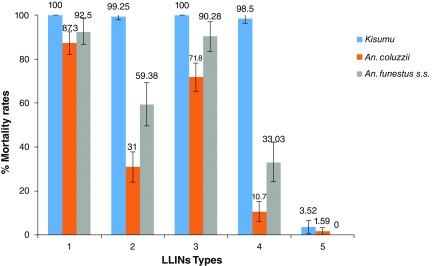
Mortality rates of resistant populations of *Anopheles funestus s.s. and An. coluzzii* and susceptible strain ( *Kisumu*) *i*n experimental huts. LLIN - Long-Lasting Insecticidal Nets.

***Mortality***. All treated nets significantly induced high lethal effect against susceptible *An. gambiae* ( *Kisumu*). In huts containing Net Type 2, 59.38% mortality rate was recorded with resistant *An. funestus s.s*. However, Net Type 1 showed a high lethal effect of 92.5% against this resistant mosquito population. Respectively, 33.03% and 90.28% mortality rates were recorded in the huts containing Net Type 4 and Type 3 ( Figure 6). Consequently, the overall killing effect offer by Net Type 1 was significantly higher than Net Type 2 against resistant *An. funestus s.s*. ( Table 6). Same thing with Net Type 3 in comparison to Net Type 4. The same trend was observed against resistant *An, coluzzii*, where very low overall killing effect was provided by Net Type 2 (28.49%) compared to Net Type 1 (73.65%). Mortalities rose from 10.7% to 71.8%, when resistant *An. coluzzii* were released in huts containing respectively Net Type 4 and Type 3. Consequently, high overall killing effect was provided by Net Type 3 against this *Anopheles* specie compared to Net type 4 ( Table 6). A combined pyrethroids-PBO Net Type 1 and Type 3 was found to demonstrate a greater efficacy against these resistant mosquito populations.

## Discussion

This study aimed to assess the response of resistant *An. funestus s.s.* from Benin to pyrethroid treated nets (current LLINs) and to combined PBO + pyrethroid nets for improved control of resistant populations of malaria vectors.

### Bio-efficacy of selected LLINs types

Results obtained from the response of susceptible mosquitoes ( *An. gambiae Kisumu*) to treated nets showed that pyrethroid and pyrethroid + PBO treated nets remained effective for controlling susceptible *Anopheles* mosquitoes. It was also observed that the bio-efficacy of nets treated with deltamethrin only (Type 2) was significantly lower when we compared the recorded mortality rates from the cone test in the resistant populations of *An. funestus s.s*. and *An. coluzzii* and the susceptible strain *Kisumu.* These observations further confirm the high pyrethroid resistance observed in both malaria vectors in Kpomè like in others localities of Southern Benin ^17, 21^. A more recent study conducted across a South-North transect of Benin, revealed that more than 50% of *An. gambiae* mosquitoes are unaffected by lethal effects of the current form of Net Type 2 ^43^. However, in the Ivory Coast, this net was effective against *An. gambiae s.s*. ^44^. When resistant mosquitoes were exposed to the combined deltamethrin-PBO (Net Type 1), the mortality rose from 56.67 to 95.77% and from 34.67 to 69.54% for respectively *An. funestus s.s.* and *An. coluzzii*. This finding showed the important involvement of P450s genes in observed pyrethroids resistance in this study and confirms also the results of synergist bioassays test performed with these same resistant mosquitoes as almost all individuals were dead when they were exposed to PBO and immediately after to deltamethrin.

Similarly, significantly lower mortality of *An. funestus s.s*. in the presence of current permethrin treated Net Type 4 was observed compared to the combined Net Type 3 (permethrin + PBO). The loss of bio-efficacy of this current Net Type 4 was also demonstrated in Malawi, Mozambique and Democratic Republic of Congo, where recorded mortality rates of *An. funestus* to Net Type 4 in this study were respectively 3%, 20% and 34% ^12, 13, 20^. The study conducted in Benin in 2013 demonstrated the efficacy of combined permethrin-PBO net (Olyset plus) against resistant *An. gambiae* ^25^. Surprisingly, only 25.83% of *An. coluzzii* was affected by lethal effect of this net in this study. It could probably due to the presence of other mechanisms involved in multi-resistance of *An. coluzzii* from Kpomè like *kdr* mutations ^30^. This result supports the relatively low mortality (69.67%) obtained from the synergist test when we pre-exposed *An. coluzzii* to PBO before to permethrin. Therefore, a combined permethrin - PBO net does not provide a solution to pyrethroid resistance with *An. coluzzii* from Kpomè, Southern Benin.

Tunnel test performed on the all net Types used in this study confirmed the reduced bio-efficacy of only pyrethroids treated nets, showing a decrease in their effectiveness in areas of high resistance. This observation could be related to the resistance selection pressure generated by the use and misuse of the same class of insecticides for malaria vector control in public health and for pest control in agriculture ^21, 45, 46^. Indeed, reduced repellent effect of Net Type 2 against wild resistant *Anopheles* mosquitoes compared to high repellent effect against *Kisumu* strain could be as a result of their resistance nature. However, crossing of mosquitoes through Net Type 1was highly inhibited for each resistant population, even for susceptible strain, penetrating the compartment C2 of the tunnel containing Net Type 1. Nevertheless, deltamethrin alone used for the treated net (Type 2) continues to have moderate performance against resistant *Anopheles* mosquitoes in terms of reducing human and malaria vector contact and also blood feeding rate.

Crossing rates with Net Type 1 were earlier described by N’Guessan *et al*., ^27^, working with *An. gambiae* VKPER a strain originate from Kou valley in Burkina-Faso even after 20 washing times, same elevated crossing rates were still recorded. We made identical observations for *An. coluzzii* in this study, with mosquitoes being no more able to pass through the net section of Net Type 3 comparing to the Net Type 4.

Similarly, *An. funestus s.s.* penetration rate recorded in presence of Net Type 4 (66.67%) was significantly higher than that of Net Type 3 (5.57%) ( *χ2* = 42.727; P < 0.0001). These observations suggest the ability of resistant mosquitoes to withstand the excito-repellency effect of LLINs and penetrate impregnated bed nets thus, feed on humans raising concerns about the duration of LLIN effective life. This high passage rate recorded with Net Type 4 could be due to the decreasing repellent effect of permethrin, which was previously pointed out with *An. gambiae* ^9^.

But the highest mortality recorded with this Net Type 4 [ *An. funestus s.s.* (71.25%) and *An. coluzzii* (45.91%)] is in line with what was reported by Darriet *et al*., ^47^ showing that bed nets treated with pyrethroids (permethrin and deltamethrin) remained effective even in areas where *An. gambiae s.s.* are resistant to these insecticides. This paradox is due to the behavioral changes in the resistant mosquitoes; they were less repelled by the insecticide, remained on the only pyrethroid-treated material for longer periods, and thus received a higher dose of insecticide leading to the death of the mosquito ^48^.

However, the mortality rates in both resistant *Anopheles* populations were significantly high in presence of Net Type 3 than Type 4 ( *χ2* =12.048; P = 0.0005). With Net Type 3 in which PBO is applied, only about 4% mosquito was able to survive the exposure. Interestingly, the same observations were found for Net Type 2 and Type 1 against both resistant mosquito populations. This test gave a slightly synergistic action of PBO on the roof of Net Type 1 with a mortality of 98% for *An. funestus s.s.* compared to the side net (92% mortality).

Concerning the control untreated Net Type 5, blood feeding was inhibited more by both Net Type 2 and Type 1 against resistant *An. funestus s.s.* This is similar to previous studies with permethrin resistant *An. gambiae s.l*. which showed that 100% blood feeding inhibition with unwashed PermaNet 3.0, a deltamethrin-PBO combination net ^27^. Blood feeding inhibition rates of resistant *Anopheles* mosquitoes with Net Type 4 were significantly lower than Net Type 3( *χ2* = 7.793;P = 0.0052), suggesting the decreased potency of this standard Net Type 4 in area where pyrethroids resistance is already spread ^9, 21, 22, 49, 50^ but overall, the effectiveness of LLINs treated only with insecticides seems to be significantly lower compared to that of nets treated with insecticides and PBO.

### Efficacy against multi-resistant mosquito strains

Experimental huts evaluations conducted in this study showed that all treated nets induced significant exophily rate ranged from 50 to 73% relatively compared to untreated net at 30% against susceptible *An. gambiae Kisumu*. This result indicates that the current pyrethroid only treated nets continue to exert strong repulsive action on the *Anopheles* susceptible strain. There was no significant difference between the low induced exophily rates in *An. funestus s.s.* and *An. coluzzii* in the hut containing Net Type 2 and Type 1 ( *χ2* = 1.837; P = 0.1753). This observation could be due to the naturally high endophilic behavior of this *Anopheles* species ^51^. Contrary, there was a significant difference between induced exiting rates of Net Type 4 and Type 3 against resistant *An. coluzzii*.

Respectively more than 40% and 60% of *An. funestus s.s*. and *An. coluzzii* survived in the hut with Net Type 2. When resistant *Anopheles* were released in the hut containing a combined deltamethrin-PBO net Type 1, less than 15% survived. However, almost all susceptible mosquito dead to the exposure with this net. Low lethal effect of Net Type 4 was observed with the resistant strain of *Anopheles* compared to Net Type 3. This result correlates with those reported by Malima *et al*. ^52^ where the recorded mortality of *An. funestus* was 71.6% against nets treated with permethrin only. Survival rates of these mosquitoes in the huts suggest that the protective nature of currently used net Type 4 in Benin is compromised, as previously reported ^53^. These semi-field controlled experiments confirmed the results from laboratory phase I evaluations and displays faith in combined pyrethroids-PBO nets, despite the multiple resistance mechanisms present in these mosquito species ^4, 19, 20, 21, 54– 56^. However, it is necessary to further investigate the impact of these multiple mechanisms on the efficacy of nets treated with pyrethroids only against *An. funestus s.s.*

A combination of the synergist PBO to pyrethroids made treated nets more efficient as PBO acted both as a metabolic enzyme inhibitor and as an adjuvant through its effect on enhanced cuticular penetration of deltamethrin ^57^. The fact that these new generation nets (Type 1 and Type 3) were able to inhibit blood feeding more than current nets (Type 2 and Type 4), could suggest their capability to confer high personal protection against resistant mosquito biting.

Studies conducted in Benin and other African countries showed a loss efficacy of pyrethroids treated net against *An. gambiae* ^9, 23, 50, 58^. This research has demonstrated that the efficacy of a combined pyrethroids-PBO nets on resistant malaria vector populations could be a promising strategy against pyrethroid resistance populations of *Anopheles* as previously highlighted ^20, 25, 27, 28, 43, 59, 60^. This study further confirms a role of oxidases in pyrethroids resistance of *An. funestus* and the need to develop nets combining pyrethroid and synergist against pyrethroid resistant malaria vectors ^25, 33, 40, 61^. Nevertheless, several other studies have been conducted on the insecticide resistance management of *Anopheles* especially *An. gambiae,* using non-pyrethroid insecticides alone or in mixture of pyrethroids ^62– 67^. These studies also provided relatively good pointers for management of resistant mosquitoes but the problems with non-pyrethroid ingredients are human toxicity and their irritant effect ^62, 68^. A more recent study conducted by Malima *et al*. ^69^ in an area where *An. funestus* is resistant to pyrethroid, showed that even when non-pyrethroid insecticide-treated nets with durable wall lining (ITWL) are used, this cannot guarantee up to 50% protection against resistant *An. funestus*.

## Conclusion

Pyrethroid resistance in the major malaria vectors *An. funestus s.s*. and *An. coluzzii* in Kpomè is high, and is likely to limit the impact of currently used LLINs. This study showed that the use of new generation bed nets could provide additional protection and reduce malaria burden in endemic environments. This study is of importance to Malaria control programs for improved control of pyrethroids resistant malaria vectors in Benin.

## Ethical considerations

Approval was obtained from the ethics review boards of the International Institute of Tropical Agriculture (IITA) ref.PJ/CC5339. All volunteers recruited to sleep in the experimental huts gave written and verbal consents. Chemoprophylaxis was provided to volunteer prior to the hut studies.

## Data availability

All data generated and analyzed during this study will be included in the published article. Raw data are available from Open Science Framework. Dataset 1: Experimental huts trial of the efficacy of pyrethroids/piperonyl butoxide (PBO) nets treatments for controlling multi-resistant populations of Anopheles funestus s.s. in Kpomè, Southern Benin. http://doi.org/10.17605/OSF.IO/3YRMS.

The data is available under a CC0 1.0 Universal License
