# Supplementary material for: Experimental huts trial of the efficacy of pyrethroids/piperonyl butoxide (PBO) net treatments for controlling multi-resistant populations of Anopheles funestus s.s. in Kpomè, Southern Benin
Source: Wellcome Open Res. 2018 Jun 13;3:71. [Version 1] doi: 10.12688/wellcomeopenres.14589.1 (PMC6113884; doi:10.12688/wellcomeopenres.14589.1)
Supplement: Peer review history

# Peer review: Referee response for version 1

**DOI:** 10.21956/wellcomeopenres.15884.r33682

**Published:** 2018-08-28

**Authors:** Parfait H. Awono-Ambene

**Affiliations:** 1 Research Institute of Yaounde (IRY), Organization for the Coordination of the fight against Endemic Diseases in Central Africa (OCEAC), Yaoundé, Cameroon

General remarks:

 The paper presents key results of an operational research topic with high significance for preventing malaria transmission in the context of multiple insecticide resistance among vector populations. The study is well designed and methods used are appropriate and linked with expected outcomes of the study.

 Though, I noticed some limitations that have not been clearly sound for me (or confusing) while reading the whole manuscript.

 For example: 

- In Abstract:
  Comment 1: Key results presented do not meet with the objectives to achieve in the subsection “background”: i) “bioefficacy of current LLINs” and ii) “performance of PBO LLINs in experimental hut”. Key results on the second objective are missing.
  Suggestion 1: provide key outcomes from experimental huts (at least mortalities by type of LLINs).
- In Method section:
  Comment 2: the sequence of subsections as presented is confusing. The subsection “study design” in page 5 appears as heading for experiments in huts with a description of the five types of LLINs to evaluate. The description of such types of LLINS was made in relation with experimental huts and seems like a redundancy of the subsection “characteristics of LLINs …..” in page 4. But no confirmation if these 5 types of LLINS were different on not to those tested with cones or tunnels. The way you have calculate some key variables in tunnels (blood feeding inhibition, overall mortality), and in huts (personal protection, overall killing effect..) is not clear.
  Suggestion 2: Confirm if types of LLINs (1 to 5) described in sections "chemical analysis of …" are also those used for all the bioassays including phase I (cone and tunnel tests) and phase II (experimental huts).
  - If so (the 5 types of LLINs related as arms for all bioassay experiments), the study design may be moved after the “study area” and adjusted accordingly prior the description of “mosquito collection”, “species identification”, “susceptibility tests with insecticide paper and with PBO synergist”, “cone tests”, “tunnel tests”, “experimental huts” and “data analysis”. Under “experimental hut” subsection may consider subparts like blank assessment of hut effects and release/recapture experiments.
  - If no (the 5 types of LLINs differ between tests), clearly indicate as “experimental hut design” and keep at this position.
  - Provide formula (available in WHOPES guidelines 2013) for the estimation of key parameters of LLIN bioefficacy in tunnels (blood feeding inhibition, overall mortality) and experimental huts (personal protection, overall killing effect..).
- In Result section:
  Comment 3: All key results related to methods used have been presented. However, redundant data and information should be limited or avoided to shorter the paper, for example:
  Suggestion 3:
  - Table 1 (distribution of members of ….) in page 6 is not necessary (optional), since data have been considered in the text at the subsection “molecular speciation …”;
  - Figure 1 in page 6 does not add value since same data are considered in figure 2 in page 7. Consider only figure 2 and insert the specific data on Kisumu strain from figure 1 in the main text.
  - Table 4 and 5 can be combined in one table of blank assessment  of attractiveness and lethal effect of experimental huts (4 columns of variables per experimental hut: two for attractiveness: "overall mosquitoes" and "Anopheles" and two for lethal effect: "tested mosquitoes" and "mortality rate")
  - Check the last sentence of the third paragraph in page 8 (column on the right)…” Against resistant An. funestus s.s., blood feeding inhibition rates (removed “s”) with net type 4 (41.75%) was significantly lower than net Type 4 (against? Should be type 3) (100%). Respectively...
  - Titles of figures 2 (page 7) and 3 (page 8) may be more comprehensive:
  Figure 2. “Mortality rates of  Anopheles to pyrethroids and when combined …”
  Figure 3: “Mortality rates reported with cone tests on different types of nets using pyrethroids  resistant …”
  - Provide statistical evidences of the conclusion at the end of subsection “blank assessment of hut attractiveness” (page 9, 2nd paragraph in column on the left)..
- In Conclusion section:
  Comment 4: The conclusions of this paper are adequately supported by the results.

Probably add elsewhere the justification and pertinence for undergoing phase II in experimental huts when LIN efficacy does not meet the WHO criteria using standard methods i.e. cone test (≥ 80% mortality or ≥ 95% knock-down) or tunnel tests (≥ 80% mortality or ≥ 90% blood-feeding inhibition).

I have read this submission. I believe that I have an appropriate level of expertise to confirm that it is of an acceptable scientific standard.

# Peer review: Referee response for version 1

**DOI:** 10.21956/wellcomeopenres.15884.r33304

**Published:** 2018-07-06

**Authors:** Emmanuel Elanga Ndille

**Affiliations:** 1 Department of Medical Entomology, Centre for Research on Infectious Diseases (CRID), Yaoundé, Cameroon

This manuscript describe results of experimental experiences performed to assess the efficacy of of pyrethroids/piperonyl butoxide (PBO) net treatments for controlling multi-resistant populations of *Anopheles funestus s.s*. The study is highly interesting as deals with development of strategies of malaria control in the context of high resistance to pyrethroid observed in main vectors. The manuscript is well written and presented, however there are very few minor concerns I would like the authors to take into consideration so that their work will be more efficient.

**Comments**: 

1. The title could be modified to also take into account *An. coluzzi* as all the results of the manuscript are also presenting data from this species
2. 2 ^nd^ paragraph of the introduction, in the 5 ^th^ sentence : “resistance to insecticide ofone of ………”  replace “ofone” by “of on”
3. In the section “Methods”, on the part “WHO cone tests with the nets” authors must keep the same references format. Indeed they should replace “( *Methods in Anopheles Research, 2010*)” by a corresponding number to be uniform in all the manuscript.
4. In the text ( *Release and recapture experiment*) the sentence “Target entomological parameters monitored included: included exiting, blood-feeding inhibition and mortality” could be change by “ *target entomological parameters monitored inclided :* *(i) insecticide-induced exiting, i.e. the proportion of mosquitoes* *found in hut verandahs relative to control huts; (ii) blood-feeding inhibition, i.e. the proportional reduction in blood-feeding relative to untreated nets; and (iii) mortality, the proportion of mosquitoes killed (immediate plus delayed)*”.

I have read this submission. I believe that I have an appropriate level of expertise to confirm that it is of an acceptable scientific standard.
